# Complete Genome Sequence of Oyster Isolate Vibrio vulnificus Env1

**DOI:** 10.1128/genomeA.00421-18

**Published:** 2018-05-17

**Authors:** Parisa Noorian, Shuyang Sun, Diane McDougald

**Affiliations:** a School of Biotechnology and Biomolecular Sciences, University of New South Wales, Sydney, Australia; b ithree Institute, University of Technology Sydney, Sydney, Australia; c Singapore Centre for Environmental Life Sciences Engineering, Nanyang Technological University, Singapore

## Abstract

Vibrio vulnificus, a ubiquitous inhabitant of coastal marine environments, has been isolated from a variety of sources. It is an opportunistic pathogen of both marine animals and humans. Here, the genome sequence of V. vulnificus Env1, an environmental isolate resistant to predation by the ciliate Tetrahymena pyriformis, is reported.

## GENOME ANNOUNCEMENT

Vibrio vulnificus is a Gram-negative opportunistic pathogen and the causative agent of gastroenteritis, primary septicemia, and necrotizing fasciitis in humans. Biotype 1 strains cause most human infections and can enter the human body through open wounds when exposed to seawater or by consumption of contaminated seafood (especially raw or undercooked oysters) (1–3).

V. vulnificus Env1 is an environmental strain isolated from an oyster in the state of Louisiana in the United States in 2005 (4) and was previously designated SS109B-3B2 (5). Env1 is from a collection of diverse V. vulnificus isolates that were tested for resistance to predation by the filter-feeding ciliate Tetrahymena pyriformis. Env1 was significantly more resistant than other isolates. T. pyriformis was unable to feed on Env1 and exhibited signs of being exposed to an antiprotozoal toxin. Here, we investigate the genomic characteristics of Env1.

Genomic DNA was extracted according to the manufacturer’s instructions using the Qiagen DNeasy genomic DNA prep kit for Gram-negative bacterial cultures, and genomic DNA libraries (15 to 20 Kb) were generated by the Ramaciotti Centre (University of New South Wales, Sydney, Australia), according to the manufacturer’s instructions. The complete genome sequence of V. vulnificus Env1 was generated using the PacBio RS platform with single-molecule real-time (SMRT) sequencing with 140× coverage. *De novo* assembly of the resulting reads was performed using the hierarchical genome assembly process version 3 (HGAP.3, Pacific Biosciences). The genome consists of 4,954,048 bp with an average guanine-plus-cytosine (G+C) content of 46.7% and, like other known Vibrio spp., contains two circular chromosomes of 3,241,343 and 1,712,705 bp. There was no evidence of a plasmid in this isolate.

The sequences were submitted to the Rapid Annotations using Subsystems Technology (RAST) (6) as well as the NCBI prokaryotic genome annotation pipeline (https://www.ncbi.nlm.nih.gov/genome/annotation_prok/) (7) servers, and results show that there are a total of 4,378 coding sequences (CDSs) and 157 RNAs predicted. Approximately 28.4% of the CDSs were annotated as hypothetical proteins. The majority of the CDSs were related to functions in subsystems, including amino acids and derivatives (515 genes), carbohydrates (456), protein metabolism (310), and the synthesis of cofactors, vitamins, prosthetic groups, and pigments (303 genes). There were 9 genes related to prophage, 52 genes to iron acquisition and metabolism, 164 genes to stress responses, and 92 to virulence, disease, and defense, as determined by the SEED viewer (an annotation environment that detects and compares genes between functional subsystems from groups of genomes) (8).

An in-depth comparison between the grazing-resistant Env1 and V. vulnificus strains CMCP6 and Yj016, which are sensitive to grazing by T. pyriformis, revealed that Env1 harbors novel virulence genes, for example, a putative internalin, putative Rhs-related proteins, an ankyrin protein, and a type 1 secretion system-associated agglutinin RTX. In a sequence-based comparison, 387 genes were annotated as hypothetical that had identity matches of less than 30% with CMCP6 and YJ016. In addition, 22 open reading frames (ORFs) were annotated as mobile elements with the same criteria.

### Accession number(s).

The complete genome sequence of V. vulnificus Env1 has been deposited in the GenBank database under the accession numbers CP017635 for chromosome I and CP017636 for chromosome II.
